# Primary healthcare services in the rural Eastern Cape, South Africa: Evaluating a service-support project

**DOI:** 10.4102/phcfm.v12i1.2207

**Published:** 2020-03-30

**Authors:** Angela A. Morris-Paxton, Stephen Reid, Rose-Marie G. Ewing

**Affiliations:** 1 Drug Utilisation Research Unit, Department of Pharmacy, Faculty of Health Sciences, Nelson Mandela University, Port Elizabeth, South Africa; 2 Primary Healthcare Directorate, Faculty of Health Sciences, University of Cape Town and Groote Schuur Hospital, Cape Town, South Africa; 3 Donald Woods Foundation, Vincent, East London, Eastern Cape, South Africa

**Keywords:** clinical outreach services, Eastern Cape, NGO, primary healthcare, rural areas

## Abstract

**Background:**

In 2012, 38% of the South African population resided in the rural areas of the country. The professional healthcare services are concentrated in the urban areas, resulting in an imbalance between urban and rural healthcare services.

**Aim:**

The aim of this study was to evaluate the use of a non-governmental organisation (NGO)-supported mobile healthcare service in a remote area.

**Setting:**

Eastern Cape Province in South Africa.

**Methods:**

The walking distance between the community and the nearest fixed government healthcare service was evaluated and compared with the recommendations of World Health Organization (WHO). Services provided to people visiting the mobile community service were recorded, and descriptive data were analysed and compared with the anonymised patient records of the nearest fixed service clinic.

**Results:**

Of the 30 outreach points served by the NGO, 24 points were at a distance more than the WHO-designated walking distance and 11 points were more than twice the WHO-designated distance from the perspective of fixed clinic. The average headcount per annum of the outreach NGO mobile clinics exceeded those of the fixed Department of Health (DoH) clinics by an average of 250 patients per clinic session. The increase in services was also noteworthy, with a mean differential of 1774 services per annum for the same day above that of the DoH clinics.

**Conclusion:**

Mobile services could make a difference to the utilisation of essential healthcare facilities. The provision of augmented NGO-led mobile clinical outreach services and joint government–NGO partnerships holds possibilities for improving healthcare for those living in remote rural areas.

## Introduction

The concept of health for all incorporates the social environment, economics, national politics and policies that drive the health user and provider practices.^1^ Funding and provision of health services in any country may be government-funded, but global health policy goes beyond national policies and tends to focus on vulnerable groups of people, regardless of where they may reside.^2^ The truly global perspective is the concept of health as a human right for all people worldwide.^1,2^ Since 2008, a change in perspective of what constitutes health recognises that it is not just the absence of disease but also encompasses the ability to live well and adapt to one’s environment.^3^ Universal health coverage as defined in the World Health Report of 2010 means that all people, regardless of where they reside, have an inherent right for adequate and reliable healthcare provisions.^4^

As of 2010, half global population was living in remote and rural areas.^4^ As of 2012, in spite of increase in urbanisation, 38% of the South African population resided in the rural areas of the country.^5^ Professional healthcare services, however, are concentrated in the urban areas.^4,6^ This results in a severe imbalance between urban and rural services, nationally as well as internationally. Socio-economic inequality affects many regions of the world, including South Africa, a situation exacerbated by inequality in access to healthcare services.^7^ The World Health Organization (WHO) report of 2010 acknowledges the fact that governments alone may not be able to provide adequate healthcare services for the needs of their respective populations.^4^ Non-governmental organisation (NGO)-supported mobile outreach primary healthcare services based within remote rural areas offer the possibility of successfully augmenting existing facilities in addition to contributing to the existing information with respect to the needs of the local area population.

The right of access to health services, regardless of geolocation, is a non-negotiable human right; however, the type of services, the extent of health service delivery and non-negotiable minimum core standards are not standardised internationally.^8^ The Sustainable Development Goals (SDGs) are to ensure healthy lives and promote well-being for all and are implemented according to each country’s means and requirements.^9^ The SDGs have one overarching goal for health: to ‘ensure healthy lives and promote well-being for all at all ages’.^10,11^ The SDG for health is as much a product of social stability and progress as it is an outcome of social stability and adequate healthcare provisions.^10^ Improvement in health is seen as an improvement in human capital, and social and economic development, which further improves health and well-being.^11^

### Rural health provision in South Africa

Section 27 (1) (a) of the Constitution of the Republic of South Africa (Act 108 of 1996) guarantees the right of access to healthcare for all, and Section 27 (3) states that no one should be refused emergency medical treatment.^12^ South Africans, however, do not have equal access to healthcare, and the disparity in access accentuates poverty and inequality in the country.^13^ The poorest members of the society often live in the remotest areas, with the least access to healthcare services, which, additionally, often do not meet the expected national standards.^13^ Despite gains made in the country as a whole for access and quality of healthcare services, these gains have not been made across the board.^14^ The South African government recognises that policies used to provide healthcare services to the nation require a significant change. The South African primary healthcare re-engineering process, therefore, is grounded in a population-based system, aimed at strengthening the services provided to marginalised communities.^15^

### Rural health provision in the Eastern Cape

The Eastern Cape province of South Africa, specifically its northeastern area, known as the wild coast, encompassing the district of Mbashe, is the most deprived area in the country with a disproportionate burden of unemployment, poverty and disease.^16^ The area falls below national and regional standards for clean water, employment and access to healthcare services.^16^ The Eastern Cape covers 13.8% of the total area of the country and is home to 12.7% of the population, which utilises 10.2% of the domestic electrification and 6.5% of the domestic piped water,^17^ both of which are below the expected per capita provision.^17^ The incidence of infectious and chronic diseases, as well as malnutrition, is higher than the national average; and the coverage of immunisation and healthcare service delivery is the lowest.^18^ This situation leaves a significant gap between the needs of the population and the services provided.^18^ Better health is often an outcome of improved socio-economic conditions, and the more impoverished communities are the most vulnerable mass to diseases.^19^ Conversely, health is also a precondition to sustainable development.^19^

### Gaps in service provision research

Even with the required resources to service a rural population, knowing who to serve, with which kind of intervention, would enable the service providers – governmental or non-governmental – to make the best use of their resources. Incomplete or non-existent vital event recording and insufficient health service registration pose challenges for the provision of adequate health services to rural areas.^20^ Data used to support health service provision are generally taken from the government health facilities. The statistical analysis of such data may miss the population who find it difficult to access services.^20^ The more advantaged are often over-represented in health research, with hard to reach populations less visible in the statistical analysis of healthcare provision, which affects the placement of resources.^21^ The WHO has identified a need for national and regional research to ensure that relevant information is available for relevant and timely responses to needs.^22^ Non-governmental organisations may have to assist in bridging the gap not only between the population needs and service provision^4^ but also in the data collection and analysis of requirements.^23^

### A South African case study

In South Africa, rural and urban populations face differing health challenges.^24^ Non-communicable diseases (NCDs) and obesity have more commonly affected urban communities, whilst infectious diseases and undernutrition have historically affected the rural community. The gap between these in terms of lifestyle conditions and transitional diets, however, is now closing.^24^ As health and socio-economic status are so closely linked, the adverse effect of ill-health in rural areas increases the socio-economic disparity, which further increases the likelihood of ill-health, especially if this is related to nutrition.^7,25^ Rural population tend to have higher levels of certain diseases, predominantly because of socio-economic conditions exacerbated by lack of healthcare resources or the means to access the healthcare resources available, resulting in significantly poor levels of health.^26^

Even if free healthcare is available, transport costs and distance to travel often affect to have timely treatment.^27^ Weathering and lack of repair of rural coastal roads make for poor public transport options to villages and towns with fixed community health services. There is no railway line, and public and private transport service is rare, expensive and dependent on road and weather conditions, which are not conducive for the use of motorbikes or even bicycles. Only few people drive as most cannot afford a private four-wheel vehicle, hence walking and donkey carts are the most common means of going from one place to another. In the Mbashe district being evaluated, one has to cover long walking distance to reach fixed government health clinics. According to the findings of this research, an average walking distance of 9 km has to be covered to reach any form of health service. With change in local demographics, predominantly older people taking care of children but experiencing the health-related problems of both ageing and poverty-related malnutrition,^25,26,28^ for them these long walking distances are untenable. These changing demographics of people in rural areas have to be taken into consideration concerning adjunct service provision, as in the supportive primary healthcare screening for infectious and chronic disease (such as hypertension, diabetes, obesity, stunting and malnutrition).^29^

As identified in 2005, factors that could improve health include having a regular water supply, provision of sanitation services and improvement in the population’s knowledge of proper sanitation and hygiene.^30^ The rural Eastern Cape, in particular the rural Wild Coast area, has lagged behind the rest of the country in respect of equal access to water, sanitation and healthcare services.^16^ In 2010, however, a new policy for the revitalisation of primary healthcare in the Eastern Cape was initiated. This new initiative responded to good international practice and followed through with the implementation of national primary healthcare plan.^31^ This policy was implemented in four areas, one of which directly affects the region under review, that of King Sabata Dalindyebo, which comes within the OR Tambo area of the rural Eastern Cape.^31^ Under this policy, one of the primary areas of focus of Health Minister was enhancing the effectiveness of health system.^31^ Additionally, the Integrated Development Plan of Mbashe Municipality, although not directly affecting health services, was expected to have an indirect positive effect on the health of area residents. The envisioned benefits were because of improvement in sanitation and waste removal, provision of clean water, and sports and recreation facilities that could promote healthy physical development.^32^

### Implementation of an augmented non-governmental organisation service

Such improvements, notwithstanding access to health services in rural areas, as well as the means to deal with the challenges of access to and payment for transportation and absentee parents of children living in rural areas, all affect the uptake of health services, even if these are available. Government and NGO partnership has worked well in the rural areas of Africa if both sides of this partnership are committed to the process and the partnership is tailored to the requirements of the community.^33,34^ There is evidence that such collaborative services lead to improvements in healthcare provision and delivery.^33,34^ This is especially the case for the areas of both conflict and extreme poverty and where provision of public health services has entirely broken down to reach rural and remote regions.^34^ In such areas, only independent NGOs provide the health services available.^34^ In the Eastern Cape province of South Africa, NGOs in agreement with local authorities have made health service partnerships to enhance the provision of local rural healthcare.

A case in point is the rural area surrounding Mbashe and the King Sabatha Dalindyebo district of Mbashe in the rural Eastern Cape. Donald Woods Foundation (DWF), an NGO, provides adjunct health service in these areas. This service is provided to remote rural village inhabitants living at a distance from central government health clinics, and includes assessment services, immediate remedial treatment and referral to fixed government primary or secondary healthcare services for both older population of the community and children. The DWF mobile tented clinical outreach service focuses on screening, onward referral, treatment of minor ailments and emergency medication. This service supports the most overloaded government primary healthcare clinics in addition to serving some of the least accessible areas.

**FIGURE 1:**
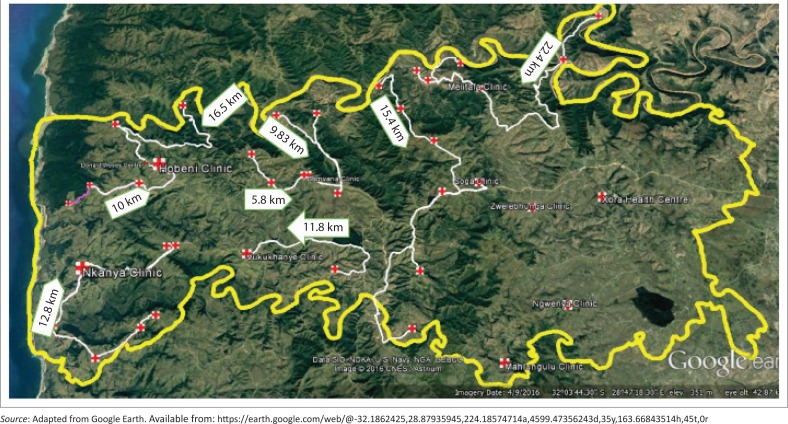
The catchment area for the study: The white arrows show the walking distances from the outermost villages to the nearest fixed clinic.

## Research methods and design

This study was an evaluation of a rural augmented mobile healthcare service. The walking distance between the central point of the community (the cluster of dwellings that constitute a ‘named village’) and government’s nearest fixed primary healthcare clinic service was assessed. This distance was compared with the WHO recommendations for walking distances to healthcare services to determine the points where mobile health services were most required. Secondly, services provided to people visiting the mobile community service were recorded in a detailed attendance log sheet to ascertain the community’s use of multiple screening and minor ailment services provided. These services included (but were not limited to) testing, emergency treatment and referral services for HIV, tuberculosis (TB), hypertension, diabetes, child immunisation and minor ailments. Descriptive data from detailed logs were analysed and compared with the anonymised patient records of the nearest fixed service clinic.

### Study settings, participant selection and sampling procedure

The study settings were the greater Mbashe area of the Eastern Cape Province, South Africa. According to the Statistics South Africa as of 2016, the total population of this area was 270 068 (125 084 males and 144 984 females); there were 56 995 households, of which 39 689 were housed in traditional dwellings with a poverty density of 44.1%.^35^ All those who visited the outreach clinics comprised the sample population, and the comparative clinic data were extracted from anonymised entries in the patient attendance records of selected Department of Health (DoH) clinics.

### Data collection and analysis

A four-wheel vehicle was used to access remote area communities, and the distance travelled from the centre of the community to the nearest fixed service was assessed. Secondly, a detailed log sheet was used to record the number of people visiting the mobile NGO service and ascertain the services provided to them. This log data were entered into a Microsoft Excel spreadsheet. The descriptive data were analysed using the Statistica data analysis programme. The data were compared with the anonymised patient records of the nearest fixed service clinic, which would normally provide services for that specific remote area community.

### Ethical consideration

Ethical approval to conduct the study was obtained from the University of Cape Town Human Research Ethics Committee (Ref. no. R033/2016).

## Results

The designated ideal distance to walk to a primary healthcare clinic should not be more than 5 km.^36^ Of the outreach points served by the DWF, however, only six outreach points were less than 5 km from the nearest fixed healthcare facility, with the remaining 24 outreach points at more than this distance and 11 points at more than twice the designated distance (Table 1).

**TABLE 1:** Villages served by the Donald Woods Foundation clinical outreach services and their respective walking distances to the nearest fixed primary healthcare clinic.

Village name	Walking distance in kilometers
Ganizulu Summit	0.17
Gqubhuzeni Outreach Point	3.00
Nyangilizwe Junior Secondary School Outreach Point	3.66
Nqileni Outreach Point	3.99
Desi Outreach Point	4.35
Ntshingeni Outreach Point	4.91
Khohlo outreach Point	5.30
Lalini Outreach Point	5.39
Phokoloshe/Kwelomthombe Outreach Point	5.58
Mabholobela Outreach Point	5.59
Mgashe Outreach Point	6.38
Ntabozuko EFT college Outreach Point	6.79
Xuba Outreach Point	7.42
Nobangile Summit Outreach Point	7.67
Kwatshezi Outreach Point	7.88
Cwebe Outreach Point	8.10
Ngqakayi / Tsholora Point	8.26
Sirhosheni Outreach Point	8.57
Ntilini Outreach Point	9.83
Geya Outreach Poin t	10.00
Botwe Outreach Point	10.60
Folokhwe Outreach Point	11.00
Mcelwane Admin Area Outreach Point	11.10
Ngqatyana Outreach Point	11.80
Mgojweni Outreach Point	12.80
Mcelwane Outreach Point	15.40
Mbelu Outreach Point	16.50
Riverview Outreach Point	17.10
Qinqana Outreach Point	18.20
Xobo Outreach Point	22.40

For four communities, to reach the outreach clinic was hampered by a river crossing, requiring a rowing boat to cross the river.^1^ The areas served are shown in Table 1 along with the relevant walking distance from outreach points to the nearest fixed government clinic. The average walking distance could only be calculated from mobile outreach clinics to the nearest fixed government health service clinic; however, the authors appreciate that individuals had to walk to mobile outreach points from their homes. This distance could have been anything from a few hundred meters to over 3 km. Given the number of people visiting mobile outreach clinics, it was not practically feasible to measure each person’s walking distance from their home to the mobile outreach clinic point.

Table 2 shows the details of 103 clinical outreach services that were conducted from the second half of 2014 to the second half of 2017. These were serviced by a mean average of 16 staff per clinic (actual figure 15.74; range 5–33), conducting an average of 429 services (actual figure 429.32; range 13–1329) for an average of 185 patients per clinic session (actual figure 185.16; range 10–471) and delivering an average of two services per patient (actual services delivered 2.39; range 1.00–4.10), with an average of 13 patients (actual figure 12.66) per staff member (range 1.0–61.20).

**TABLE 2:** Summary of the clinical outreach service personnel, patients and services for the 3-year period of research.

Personnel, Patients and Service	Descriptive Statistics (Outreach Reports Data Collection – 36 months – June 2017)				
	Valid N	Mean	Minimum	Maximum	s.d.
Total-DoH-Personnel	103	4.59	0.00	14.00	2.21
Total-DWF-Personnel	103	8.97	3.00	29.00	3.82
Total-OPS-Personnel	103	2.17	0.00	18.00	4.20
Total-Personnel-All	103	15.74	5.00	33.00	5.24
Total Serv ice	103	429.32	13.00	1329.00	239.60
Clinic-Patient-Headcount	103	185.16	10.00	471. 00	100.87
Av Patient per Staff	103	12.66	1.00	61.20	8.50
Av-Ser-PP	103	2.39	1.00	4.10	0.71

Note: Figures are given in whole units for personnel and services.

DoH, Department of Health; DWF, Donald Woods Foundation; OPS, Other (volunteer) Personnel Services; Av-Ser-PP, average service rendered per patient; s.d., standard deviation; NGO, non-governmental organisation.

*N* = NGO’s 103 tented clinics.

Tables 3 and 4 compare the NGO and DoH clinic services given on the same day for three specific areas. Considering the full 3-year period of research for the matched same-day clinical outreach and DoH clinic visits, the average headcount of the outreach DWF mobile clinics per annum exceeded that of the fixed DoH clinics by an average of 250 patients per single clinic session. In addition to increase in headcount, increase in services provided was also noteworthy with a mean differential of 1774 services per annum for the same day, this exceeding that of DoH clinics. The average service given per person was also 2.94 times higher in the outreach clinics than that provided by fixed DoH clinics for the same period. It appears that the clinical outreach service was the preferred service for patients in remote outlying areas despite the availability of DoH services. Apart from the convenience of shortened distance and greater accessibility to the NGO service, the average of services provided per patient was notably higher, indicating greater efficiency and better use of resources.

**TABLE 3:** Matched sample of clinical outreach service (Bomvana, Hobeni and Melitafa areas), patient headcount and total number of services with average service per patient for the 3-year period of research.

Patients and Average Service	Descriptive Statistics (comparative – 36months condensed-Aug 2017)				
	Valid N	Mean	Minimum	Maximum	s.d.
Total Serv ice	3	3323.67	1512.00	5789.00	2212.16
Clinic-Patient-Headcount	3	1298.67	511.00	2094.00	791.53
Av -Ser-PP	3	2.60	2.07	2.96	0.47

Note: Figures are given in whole units for the number of services. Av-Ser-PP, average service rendered per patient; s.d., standard deviation; NGO, non-governmental organisation.

*N* = NGO’s three tented clinics on the same day as the fixed government district clinic.

**TABLE 4:** Matching sample of the Bomvana, Hobeni and Melitafa Department of Health clinics’ headcount for the 3-year period of research with that of sample outreach clinic’s patient headcount and total number of services provided with average service per patient on the same day for the same period of research.

Patients and Average Service	Descriptive Statistics (comparative – 36months clinic same-date-Aug 2017)				
	Valid N	Mean	Minimum	Maximum	s.d.
Total Service DoH	3	1550.00	1301.00	2035.00	420.07
Clinic-Patient-Headcount	3	1048.33	828.00	1299.00	236.96
Av-Ser-PP	3	1.48	1.29	1.57	0.16

Av-Ser-PP, average service rendered per patient; s.d., standard deviation; DoH, Department of Health.

Figures are given in whole units for the number of services.

*N* = Three fixed government clinics on the same 3 days as the tented clinical outreach service.

## Discussion

International NGOs such as Medicines Sans Frontiers, the International Red Cross/Red Crescent and the Gift of the Givers often work in areas of crisis and conflict. Locally operating internationally funded NGOs, such as The United States Agency for International Development (USAID), often focus on specific and critical needs such as HIV and TB testing and treatment, as opposed to ongoing primary care at community level. Public–NGO collaboration in the healthcare sector has increased in low- and middle-income countries as a means of improving healthcare.33 This type of organisational collaboration works with government department, which provides some salaried local staff as a support working with locally recruited NGO workers receiving an NGO-funded stipend. Medications may be provided and/or funded by the government, but transportation of staff and supplies is often the responsibility of NGOs transport and logistical department. Advantages of such collaborations are that locally staffed NGOs, with external expert training, often build trust to have a better understanding of both needs and challenges of local population.33 Over the longer period, the intention of the government could be to take over the services of NGO once the local staff are trained and funding for the services is secured; however, the public sector may not be able to continue services alone once the NGO has left the region.34

Understanding the spatial distribution of disease is necessary for the provision of adequate services and facilities in rural areas.^37^ Walking distance to the outreach clinic in this article varied considerably from less than 1 km to more than 22 km, with in most cases, this being more than the WHO-recommended distance of less than 5 km between patient’s dwelling and the nearest primary healthcare facility.^36^ The utilisation results indicate that for rural communities having very limited access to vehicles, mobile clinical outreach services are much more appropriate and accessible than fixed healthcare government facilities. Distance is, however, not the only determining factor in choosing healthcare facilities, as other factors, such as the nearest healthcare facility to one’s place of work, education, other social services and shopping facilities, may also be determining factors in choosing access to primary healthcare.^13,36^ Other possible factors in preferring healthcare services may be the possibility of overcrowding of clinic facilities, long waiting period, and the number and type of services offered.

Furthermore, road conditions and cost of travel come into play when determining the feasibility of travelling to the nearest healthcare facility. In this respect, the nearest service available may not be the most accessible one.^13^ Topography, such as river-crossings in the Mbashe area, presents additional challenges and could become the deciding factor for elderly and those with small children in both choosing and visiting a healthcare service.

### Limitations

The population served in each area was the same for both government’s DoH clinics and NGO-led mobile outreach clinics; however, the NGO-led outreach clinics served those living on the outer edges of catchment areas. Hence, for such rural populations, the mobile NGO outreach clinics were more convenient and easier to reach. This could have put demand pressures on NGO-led mobile clinics, although this was hard to measure as there tended to be a higher population density surrounding government clinics, with schools and other facilities. The skill levels of both NGO staff and government staff were comparable as all staff had to be registered with the Nursing Council of South Africa or Health Professional Council of South Africa (HPCSA), but the NGO had more resources to recruit and pay their staff; in addition, they were able to recruit retired experienced government staff. The authors acknowledge that initially there may have been a ‘novelty’ factor in the provision of NGO-led services; however as the services ran for many years and the study was conducted over a 3-year period, this was likely to be a minor factor to visit mobile NGO-led outreach clinics. The full expenditures of the NGO-led services were not ascertainable as the accounts were confidential and not shared with researchers.

The study has further limitations in making an adequate full comparison of all NGO services with that of formal government fixed clinics. Insufficient staffing and computers and intermittent electric supply were some of the factors resulting in inadequate recording of patient details and service provision in the government sector. Such situations are not unusual in the public sectors in low- and middle-income countries, and similar challenges were found to hamper NGO provisions and evaluation of rural healthcare services in Ghana^33^ as well as Sudan.^34^

### Recommendations

Further evaluation of NGO-led clinical outreach services could add to our knowledge about communities, their needs of type of services and where these communities are located. Such information could reduce wastage of expenditure and increase the effectiveness of available resources.

## Conclusion

Distance is only one aspect of access to healthcare to be considered in rural communities. Other aspects include complementary services, condition of roads, access to vehicles, and local topography and community acceptability of services. Mobile clinical outreach services could make a measurable difference in utilisation of essential services. Furthermore, provision of augmented NGO-led mobile clinical outreach services and joint government–NGO partnerships hold possibilities for improving healthcare facilities for those living in remote areas. Essential screening services offered at remote outreach points add value to the overall screening and referral services in rural areas with limited resources. The extent of utilisation by the community illustrates the need for NGO involvement in remote areas for provision of healthcare services. The ability of such NGO-led healthcare services to provide for the needs of the rural population appropriately, as required, may be a critical factor in filling the service provision gaps found in rural areas. As this investigation covered a very specific NGO–government health provision partnership, further research could clarify the broader role of NGOs in providing health services.

## Footnotes

1 The four highlighted areas depict villages where there is no bridge to cross the river to reach the clinical outreach service; residents are required to use a rowing boat to access the outreach clinic tents.
